# The challenges of responding to misinformation during a pandemic: content moderation and the limitations of the concept of harm

**DOI:** 10.1177/1329878X20951301

**Published:** 2020-11

**Authors:** Stephanie Alice Baker, Matthew Wade, Michael James Walsh

**Affiliations:** City, University of London, UK; The Australian National University, Australia; University of Canberra, Australia

**Keywords:** content moderation, COVID-19, disinformation, misinformation, social media, trust

## Abstract

Social media have been central in informing people about the COVID-19 pandemic. They influence the ways in which information is perceived, communicated and shared online, especially with physical distancing measures in place. While these technologies have given people the opportunity to contribute to public discussions about COVID-19, the narratives disseminated on social media have also been characterised by uncertainty, disagreement, false and misleading advice. Global technology companies have responded to these concerns by introducing new content moderation policies based on the concept of harm to tackle the spread of misinformation and disinformation online. In this essay, we examine some of the key challenges in implementing these policies in real time and at scale, calling for more transparent and nuanced content moderation strategies to increase public trust and the quality of information about the pandemic consumed online.

Content moderation is used in a variety of contexts as a ‘governance mechanism’ to structure community participation to facilitate cooperation and civility (Grimmelmann, 2015). On social media, content moderation is typically an automated process based on machine learning and computer algorithms characterised by limited human interaction. Historically, social media companies have resisted intervening in public discourse, perceiving of themselves as impartial conduits for conversations rather than curators of content. In recent years, these companies have been criticised for not taking their influence on public discourse seriously enough with regard to issues around violence, discrimination and political interference as exemplified by the Cambridge Analytica scandal in which political groups were exposed for misusing the data of millions of Facebook users. The global spread of misinformation about COVID-19 on social media has led to an ‘infodemic’, as the World Health Organization (WHO) termed it, with platforms under pressure to respond to false and misleading information about the virus.

In an effort to increase public trust and avoid government oversight, technology companies have worked together with governments and healthcare agencies to elevate authoritative sources related to COVID-19 and to jointly combat fraud and misinformation about the virus. Most of these policies centre around the concept of harm. Twitter, for example, has broadened its definition of harm to address ‘content that goes directly against guidance from authoritative sources of global and local public health information’ (Gadde and Derella, 2020). Facebook and Instagram announced that they would ‘remove COVID-19 related misinformation that could contribute to imminent physical harm’ (Clegg, 2020), and YouTube (2020) created a ‘COVID-19 Medical Misinformation Policy’ that prohibits misinformation about COVID-19 that ‘poses a serious risk of egregious harm’ and contradicts medical information provided by the WHO or local health authorities.

While technology companies – Facebook, Reddit, Google, LinkedIn, Microsoft, Twitter, YouTube – have taken a coordinated approach to content moderation by directing users to reliable information from government healthcare agencies when they log into the service or search for related content, their approaches to combatting misinformation related to COVID-19 differ in significant ways. For example, while the controversial film, *Plandemic* (2020), which documents numerous false and misleading claims about COVID-19, was removed from Facebook and YouTube for spreading misinformation and inciting harm, shorter excerpts of the film were permitted on these sites. Twitter also allowed the film’s protagonist, Dr Judy Mikovits, to maintain her account, which she uses to promote dubious health claims to her followers. Despite these companies’ shared intention to mitigate harm, the varied outcomes of their harm policies point to the difficulties of operationalising the concept of harm into automated content moderation strategies at scale. In what follows, we elaborate on the key challenges of reducing content moderation strategies to the concept of harm in relation to the COVID-19 pandemic.

## Scientific understandings of harm are uncertain and evolving

Given the novelty of COVID-19, much remains unknown about the virus and a considerable amount of what is thought to be known could turn out upon closer inspection to be inaccurate, incomplete or based on an obsolete knowledge of the virus. Take, for example, the WHO’s (2020a) suggestion on 14 January 2020 that ‘Chinese authorities have found no clear evidence of human-to-human transmission’ of COVID-19, which turned out to be false. Likewise, the WHO’s initial guidance against the use of face masks countered much scientific advice, until the WHO updated their official guidance on the use of masks to protect against and limit the spread of COVID-19 on 5 June ‘based on new scientific findings as the epidemic evolves’ (WHO, 2020b). Issues around accuracy are confounded not only by emerging data – the notion of scientific facts as ‘moving targets’ (Scheufele et al., 2020) – but the accessibility of pre-prints not yet subject to peer review and medical papers based on unreliable data rather than randomised controlled trials, both of which raise quality issues as exemplified by now retracted papers from *The Lancet* and *New England Journal of Medicine* concerning the efficacy of the malaria drug, hydroxychloroquine, as a treatment for COVID-19. The problem social media companies face is how to engineer harm from a theoretical concept to an algorithmic practice in real time and at scale to contend with issues of uncertainty, inaccuracy and emerging data.

Conceptions of harm are particularly tenuous when used to regulate information related to causes and treatments for the virus. What currently appears to be accurate information (e.g. COVID-19 originated in a wet market in Wuhan, China) may turn out to be false. Removing conflicting claims from social media sites based on current official advice could erode public trust in scientific and medical professionals. Moreover, there is ambiguity with regard to the efficacy of treatments and preventive strategies related to COVID-19 both among and within the scientific community (e.g. claims about ibuprofen’s role as a potential treatment). While some users have been criticised for promoting immune boosting diets and supplements on social media to protect themselves from COVID-19, scientific research provides compelling evidence for the role of vitamin D to improve people’s immune system when they have a deficiency, making them less susceptible to morbidity and mortality as a result of catching COVID-19 (Grant et al., 2020). Although vitamin D is not an established treatment for COVID-19, the language around health and disease prevention is nuanced and difficult to regulate at scale.

## The concept of harm is not neutral

Social media platforms are never neutral and impartiality is impossible to implement (Gillespie, 2018). This is especially the case regarding the concept of harm, where the decision to moderate content extends beyond legal requirements. When Twitter announced in March 2020 that it would broaden its definition of harm in light of COVID-19, the revised definition included more serious examples of misinformation (e.g. specific and unverified claims that incite people to action and cause widespread panic, social unrest or large-scale disorder) to the denial of global or local health authority recommendations (e.g. denying the efficacy of social distancing measures), and the ‘description of alleged cures for COVID-19, which are not immediately harmful but are known to be ineffective’ (Gadde and Derella, 2020). Tweets from the Presidents of Venezuela and Brazil were removed for violating this policy, with tweets by world leaders subject to greater levels of regulation than those posted by average users. At the same time, a potentially harmful tweet by US President Trump claiming that ‘HYDROXYCHLOROQUINE & AZITHROMYCIN, taken together, have a real chance to be one of the biggest game changers in the history of medicine’ was permitted on Twitter; despite correlating with a 2000% increase for the anti-malarial drugs hydroxychloroquine and chloroquine in the United States between 15 to 21 March 2020 (Vaduganathan et al., 2020). While claims promoting false treatments for COVID-19, such as advocating drinking bleach or ingesting colloidal silver, are evidently harmful and more straightforward to regulate, in some circumstances official guidance from global and local health authorities designed to mitigate harm remain highly contested (e.g. school closures, lockdown and quarantine guidelines).

Policies based on harm are ideologically motivated and politically biased. By virtue of the way content moderation practices are enacted from the standpoint of the individual user, they necessarily arbitrate between competing – and often incompatible – political interests and values. This occurs at the individual, national and international level. It is exemplified by varied user perspectives on COVID-19; competing state and national Government interests in the case of the United States and the United Kingdom, where scientists petitioned against the UK Government’s delay in introducing lockdown measures, and differences between countries. For example, Sweden and New Zealand’s initial responses to COVID-19 were diametrically opposed, the former based on ‘herd immunity’ and the latter based on an elimination approach; both of which used scientific evidence to arrive at vastly different government policies. Despite some governments suggesting that their policies are ‘led by the science’, scientific views remain diverse and subject to competing interests and beliefs even within a single national context. Moreover, there are some questions that science cannot solely answer (e.g. whether to prioritise the economy or public health). The policy process is a highly contested space. Decisions about which lives to prioritise extend beyond utilitarian metrics, entering into the domain of ethics and moral philosophy. They are complex questions that transcend scientific understandings of safety and harm.

## The concept of harm can be gamed to promote uncertainty, publicity and political values

Due to the volume of information about COVID-19 shared on social media, social media companies such as Twitter have relied increasingly on users (in addition to automated technologies and moderators) to alert them about misinformation and potentially harmful content. Content perceived to be harmful is then flagged, labelled, demoted or removed. At its most basic, these techniques indicate an objection. They are part of the process of content moderation, which is subject to competing values and human interpretation. Despite being designed to mitigate harm, these practices can be gamed via algorithmic steering to produce mock outrage and publicity (Crawford and Gillespie, 2016: 420) or to promote uncertainty and doubt. This is particularly the case with disinformation campaigns, which seek to provoke discord and uncertainty in their targets rather than prove the veracity of a claim. Examples of disinformation commonly include memes, questions and personal anecdotes to sow the seeds of doubt (Baker and Rojek, 2019). During the height of the COVID-19 pandemic, strategies of this kind were evident with regard to the spread of 5G conspiracy theories with many high-profile users questioning the safety of these technologies (Baker, 2020). Despite the harmful effects of disinformation, false and misleading claims are difficult to moderate when framed as questions and personal experience rather than medical advice.

## Conclusion: moving beyond conceptions of harm

In light of the pandemic and associated ‘infodemic’, social media companies have implemented content moderation at an unprecedented speed and scale. The transmission of false and misleading information, whether shared intentionally or not, can influence beliefs and result in ‘real-world harm’. Consequently, it is understandable that the collective response of social media companies in combatting misinformation has been framed in terms of mitigating harm. However, as we have demonstrated throughout this essay, the concept of harm is neither implemented easily at speed nor scale. Engineering the concept of harm into algorithms and automated processes requires acknowledging that science is uncertain, evolving and must cope with the emergence of real-time data; that the concept of harm is not neutral and will vary from the standpoint of individuals and collectives; and that content moderation can be gamed to promote certain values, generate publicity and foster doubt. Acknowledging these challenges points to the need to move beyond removing ‘harmful’ content on the basis that it violates ‘official’ advice to considering alternative strategies of labelling and tagging posts that appear to be false and retracting these statements if they turn out upon closer inspection to be valid. In practice, this would mean only removing content advocating harmful treatments and advertising breaches for fraudulent products where there is scientific consensus that these claims pose an imminent danger to consumers (e.g. products promoting ingesting colloidal silver as a treatment for COVID-19). Misinformation of this kind could be detected via key word searches and compiled in a register that would open these claims to public audit; distinct from Facebook and Twitter’s ‘Transparency reports’, which only provide country statistics on removal requests without any insight into which claims have been removed, by whom and why. Where health claims appear misleading, yet remain uncertain, labels could be used instead to highlight that such content counters the official public health advice of a particular government or health organisation while linking to that authoritative advice. Applying these strategies would make a clear distinction between what is scientifically known to be harmful and claims that violate specific policy guidelines. By providing transparency and accounting for the complexities of implementing content moderation strategies efficiently and at scale, social media companies will not only provide more reliable content but increase public trust.

## References

[bibr1-1329878X20951301] Baker SA (2020) Tackling Misinformation and Disinformation in the Context of COVID-19 (Cabinet Office C19 Seminar Series). Available at: https://openaccess.city.ac.uk/id/eprint/24612/1/Summary%20of%20Cabinet%20Presentation%20%2808%2007%2020%29.pdf (accessed 8 July 2020).

[bibr2-1329878X20951301] Baker SA Rojek C (2019) Lifestyle Gurus: Constructing Authority and Influence Online. Cambridge: Polity Press.

[bibr3-1329878X20951301] Clegg N (2020) Combating COVID-19 misinformation across our apps. Facebook, 25 March. Available at: https://about.fb.com/news/2020/03/combating-covid-19-misinformation/(accessed 2 May 2020).

[bibr4-1329878X20951301] Crawford K Gillespie T (2016) What is a flag for? Social media reporting tools and the vocabulary of complaint. New Media & Society 18(3): 410–428.

[bibr5-1329878X20951301] Gadde V Derella M (2020) An update on our continuity strategy during COVID-19. Twitter, 16 March. Available at: https://blog.twitter.com/en_us/topics/company/2020/An-update-on-our-continuity-strategy-during-COVID-19.html (accessed 1 June 2020).

[bibr6-1329878X20951301] Gillespie T (2018) Custodians of the Internet: Platforms, Content Moderation, and the Hidden Decisions That Shape Social Media. New Haven, CT: Yale University Press.

[bibr7-1329878X20951301] Grant WB Lahore H McDonnell SL , et al. (2020) Evidence that vitamin D supplementation could reduce risk of influenza and COVID-19 infections and deaths. Nutrients 12(4): 988. 10.3390/nu12061620 PMC7352449 32492787

[bibr8-1329878X20951301] Grimmelmann J (2015) The virtues of moderation. Yale Journal of Law & Technology 17: 42.

[bibr9-1329878X20951301] Scheufele DA Krause NM Freiling I , et al. (2020) How not to lose the COVID-19 communication war. Issues in Science and Technology, 17 April. Available at: https://slate.com/technology/2020/04/covid19-misinformation-science-communication.html (accessed 1 June 2020).

[bibr10-1329878X20951301] Vaduganathan M Van Meijgaard J Mehra MR , et al. (2020) Prescription fill patterns for commonly used drugs during the COVID-19 pandemic in the United States. Journal of the American Medical Association 323: 2524–2526. 32463459 10.1001/jama.2020.9184 PMC7256862

[bibr11-1329878X20951301] World Health Organization (WHO) (2020a). Available at: https://twitter.com/WHO/status/1217043229427761152 (accessed 2 May 2020).

[bibr12-1329878X20951301] World Health Organization (WHO) (2020b) Coronavirus disease (COVID-19) advice for the public: when and how to use masks. WHO, 5 June. Available at: https://www.who.int/emergencies/diseases/novel-coronavirus-2019/advice-for-public/when-and-how-to-use-masks (accessed 6 June 2020).

[bibr13-1329878X20951301] YouTube (2020) COVID-19 medical misinformation policy. Available at: https://support.google.com/youtube/answer/9891785?hl=en-GB (accessed 1 June 2020).

